# Change of Address

**Published:** 1892-02

**Authors:** 


### CHANGE OF ADDRESS.

The office of the JOURNAL has been removed from Forty-second street and Park avenue to 311 West Fifty-ninth street, New York. The new office is at the corner of Fifty-ninth street, at the intersection of Broadway and Eighth avenue (Eighth avenue entrance to Central Park), where Veterinarians visiting New York will be welcome.

